# Meat consumption and cancer of unknown primary (CUP) risk: results from The Netherlands cohort study on diet and cancer

**DOI:** 10.1007/s00394-021-02600-5

**Published:** 2021-06-21

**Authors:** Karlijn E. P. E. Hermans, Piet A. van den Brandt, Caroline Loef, Rob L. H. Jansen, Leo J. Schouten

**Affiliations:** 1 Department of Epidemiology, GROW School for Oncology and Developmental Biology, Maastricht University, PO Box 616, 6200 Maastricht, The Netherlands; 2 Department of Research, Comprehensive Cancer Organisation the Netherlands, Utrecht, The Netherlands; 3 Department of Internal Medicine, Medical Oncology, Maastricht University Medical Center, Maastricht, The Netherlands

**Keywords:** Cancer of unknown primary (CUP), Red meat, Processed meat, Poultry, Fish, Prospective cohort study

## Abstract

**Purpose:**

Cancer of unknown primary (CUP) is a metastasised cancer for which no primary lesion could be identified during life. Research into CUP aetiology with respect to dietary factors is particularly scarce. This study investigates whether meat consumption is associated with CUP risk.

**Methods:**

Data was utilised from the prospective Netherlands cohort study that includes 1,20,852 participants aged 55–69 years. All participants completed a self-administered questionnaire on diet and other cancer risk factors at baseline. Cancer follow-up was established through record linkage to the Netherlands Cancer Registry and the Dutch Pathology Registry. A total of 899 CUP cases and 4111 subcohort members with complete and consistent dietary data were available for case–cohort analyses after 20.3 years of follow-up. Multivariable adjusted hazard ratios (HRs) were calculated using proportional hazards models.

**Results:**

We found a statistically significant positive association with beef and processed meat consumption and CUP risk in women (multivariable adjusted HR Q4 vs. Q1 1.47, 95% CI 1.04–2.07, *P*_trend_ = 0.004 and Q4 vs. Q1 1.53, 95% CI 1.08–2.16, *P*_trend_ = 0.001, respectively), and a non-significant positive association with processed meat consumption and CUP risk in men (multivariable adjusted HR Q4 vs. Q1 1.33, 95% CI 0.99–1.79, *P*_trend_ = 0.15). No associations were observed between red meat (overall), poultry or fish consumption and CUP risk.

**Conclusion:**

In this cohort, beef and processed meat consumption were positively associated with increased CUP risk in women, whereas a non-significant positive association was observed between processed meat consumption and CUP risk in men.

**Supplementary Information:**

The online version contains supplementary material available at 10.1007/s00394-021-02600-5.

## Introduction

Cancer of unknown primary (CUP) is a metastasised malignancy for which the primary tumour origin remains unidentifiable during a patient’s lifetime [1, 2]. It ranks fourth in the most common metastasised cancers in the Netherlands, and with slightly more than 1300 incident cases in 2018, CUP accounted for almost 2% of all new cancer diagnoses in that year [3, 4]. Globally, the median survival for CUP patients is only 3 months, dependent on available diagnostics as well as incidence and patient characteristics (favourable or unfavourable prognosis, 20–80%, respectively) [5–7]. For most CUP patients, curative treatment(s) may no longer be an option [8]. By assessing lifestyle factors that are potentially associated with the disease, however, it may be possible to prevent future CUP patients. Certain modifiable risk factors, such as cigarette smoking and alcohol consumption, have been linked to the development of CUP [9–12]. Nonetheless, the relationship between diet and CUP has been less well studied, and that is particularly true with respect to meat consumption [11].

The consumption of red meat and processed meat has been linked to several types of cancer in previous studies [13]. Indeed, the weight of evidence is such that the International Agency for Research on Cancer (IARC) describes red meats as “probably carcinogenic to humans”, and there is also sufficient evidence to classify processed meats as “carcinogenic to humans” [13]. Red meats are unprocessed mammalian muscle meat that contain proteins and important micronutrients, such as B vitamins, iron, and zinc [13, 14]. Processed meats, by contrast, are those meats that have been transformed through salting, curing, fermentation, smoking, or other processes so as to enhance their flavour or improve their preservation [13]. When those meats are being processed, it can lead to the formation of carcinogenic chemicals (including N-nitroso-compounds (NOC) and polycyclic aromatic hydrocarbons (PAH)) [13, 15]. In addition, the cooking of processed meat (fried, grilled, roasted, boiled and smoked), temperature and duration of cooking, type of fuel used for cooking, and proximity and direct contact with the heat source, can produce known or suspected carcinogens, including heterocyclic aromatic amines (HAA) and PAH [13, 15]. While the connection between consuming red meat and processed meat and developing cancer appears rather consistent, the connection between consuming poultry and fish and developing cancer is much less clear. Fish consumption has, however, been linked to anti-inflammatory and anticarcinogenic effects of long-chain *n*-3 fatty acids and could thus be beneficial for inhibiting carcinogenesis [16].

The IARC Monographs Working Group has evaluated the consumption of red meat and processed meat with respect to carcinogenicity to humans. Based on epidemiological evidence, it concluded that there are convincing associations between the consumption of red meat and cancer, particularly for cancers of the colorectum, pancreas and prostate [13]. In addition, the consumption of processed meat has been linked to cancers of the colorectum and stomach [13]. The 2018 Continuous Update Project Expert Report of the World Cancer Research Fund (WCRF) and American Institute for Cancer Research (AICR) concluded that the data to study the relation between poultry and cancer risk was “too low quality or too inconsistent, or the number of studies too few, to allow conclusions”. For fish consumption, they summarized a ‘limited to suggestive’ decreased risk of cancers of the colorectum and liver [17].

The relationship between meat consumption and CUP has been investigated in one Australian prospective cohort study [11]. Its authors found no association between red meat consumption and CUP risk, though they did observe a slightly increased risk between processed meat consumption and CUP risk, albeit this was not deemed statistically significant [11]. The current study assesses the association between meat consumption and CUP risk in greater depth by assessing combined groups of meats, such as red meat, processed meat, poultry, and fish, as well as individual meat items.

In addition, we investigated whether sex or cigarette smoking status influence the association between meat consumption and CUP risk, by testing multiplicative interactions.

## Materials and methods

### Design and study population

The Netherlands cohort study on diet and cancer (NLCS) includes 1,20,852 participants aged 55–69 years from 204 Dutch municipalities. The case–cohort design was applied for data processing and analysis. Cases were derived from the full cohort, while the number of person years at risk for the full cohort was estimated from a subcohort of 5000 participants who were randomly sampled from the full cohort at baseline in 1986 [18].

### Outcome measure

CUP is defined as a metastasised epithelial malignancy with no identifiable primary tumour origin after cytological and/or histological verification during a patient’s lifetime. This CUP definition only includes epithelial malignancies (ICD-O-3 M-8000–M-8570), which excludes for example sarcoma, lymphoma, mesothelioma, and melanoma.

### Follow-up

**Fig. 1:**
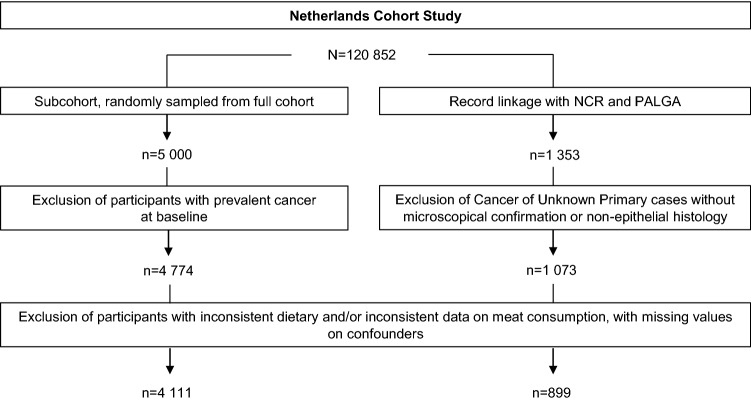
Netherlands cohort study

Cancer follow-up was established through annual record linkage with the Netherlands Cancer Registry (NCR) and the Dutch Pathology Registry (PALGA) [19]. Information regarding the site of metastasis was obtained from the NCR, but this data was only partially available and, therefore, supplementary information was retrieved from the pathology excerpts provided by PALGA. These pathology excerpts were also used to determine whether cytological and/or histological confirmed cases had been correctly categorised in the data received from the NCR. After 20.3 years of follow-up (17 Sep 1986 until 31 Dec 2006), data was available for a total of 1353 potential CUP cases, and a subcohort of 4774 participants after removing members who reported a history of cancer (except for skin cancer) at baseline. After excluding CUP cases without microscopical confirmation or non-epithelial histology, a total of 1073 CUP cases remained. CUP cases were further subdivided according to histology, according to the number of metastases (multiple metastases of the same type were counted as one metastatic site, for example, bone metastases in hip and vertebra were counted as one), according to localisation of metastasis (up to four locations), and according to survival duration. Participants were removed from the analysis if there was incomplete or inconsistent dietary data, or if there were selected confounders with missing values. As a result, 899 CUP cases and 4111 subcohort members were available for assessment (see Fig. 1).

### Questionnaire data

Participants completed a self-administered questionnaire that included detailed questions on dietary habits, lifestyle, and other cancer risk factors. A 150 item semi quantitative food-frequency questionnaire was used that concentrated on the habitual consumption of food and beverages during the year preceding baseline [20, 21]. The food frequency questionnaire had been validated against a 9 day diet record and was tested for reproducibility in the NLCS [22, 23]. The Spearman correlation coefficients for the validity of red meat, processed meat, and fish, as investigated by the questionnaire were, 0.46, 0.54 and 0.53, respectively, compared to the results of the 9 day diet record [22]. The questionnaire contained 14 items on the consumption of meat as the main meal, five items on the consumption of meat used as a sandwich filling, and three items on the consumption of fish. Meats were grouped into red meat (overall), processed meat, and poultry. Red meat included beef, pork, minced meat (beef and pork), liver, and other meats (e.g., horsemeat, lamb). Processed meat (meat items that had undergone some form of preservation with nitrite salt, fermentation, or smoking) included ham, bacon, smoked beef, pork loin roll, and other sliced cold meats (e.g., sausages). Poultry included chicken and turkey. Fish consumption was measured in relation to the main meal, lunch, or as a snack between meals.

### Statistical analysis

Person-years at risk were calculated from baseline (17 Sep 1986) until CUP diagnosis, death, emigration, loss to follow-up, or end of follow-up (31 Dec 2006), whichever occurred first. General characteristics were presented for subcohort members and CUP cases with frequencies (%) for categorical variables and means including standard deviations for continuous variables. Based on the distribution of the subcohort, participants were compared using quartiles (Q) or categories of red meat, processed meat, poultry, and fish consumption. For continuous analyses, increments of 50 g per day were used for red meat, beef, pork, minced meat, and poultry consumption, and increments of 25 g per day were used for liver, processed meat, and fish consumption.

The predefined confounders included age at baseline (years, continuous), sex (male/female), alcohol consumption (ethanol intake per day), cigarette smoking status (never/ever), cigarette smoking frequency (number of cigarettes smoked per day), cigarette smoking duration (number of years smoking), and total energy intake (kcal/day). The potential confounders included body mass index (BMI) at baseline (kg/m^2^), non-occupational physical activity (< 30 min/day, 30–60 min/day, 60–90 min/day and > 90 min/day), socio-economic status (highest level of education); diabetes (yes/no); history of cancer in a first degree relative (yes/no); and vegetable and fruit consumption (grams per day). Variables were considered a confounder if they changed the HR by > 10%. Accordingly, none of the potential confounders were included in the final model. No mutual adjustments were conducted between meat groups, as there was insufficient scientific evidence to conclude that they were related to CUP development.

Cox proportional hazards models were utilised to estimate age and sex adjusted, and multivariable-adjusted hazard ratios (HRs) with 95% confidence intervals (CIs). Standard errors were calculated using the robust Huber–White sandwich estimator to account for additional variance introduced by sampling from the full cohort [24]. The proportional hazards assumption was tested using the scaled Schoenfeld residuals [25]. In cases where the assumption had been violated, a time-varying covariate for that variable was added to the model where appropriate. Ordinal exposure variables were fitted as continuous variables in trend analyses. Wald tests and cross-product terms were used to evaluate possible multiplicative interaction between sex in relation to meat consumption and CUP risk, or between cigarette smoking status in relation to meat consumption and CUP risk. Analyses were conducted using Stata version 15. *p* values were considered statistically significant if *p* < 0.05.

Three sensitivity analyses were conducted, the first of which was restricted to histologically verified CUP cases only, since it is more likely that those cases had undergone extensive diagnostic investigation(s) to rule out the primary tumour origin. For those patients who received cytological verification alone, other factors may have played a role in the decision to refrain from further diagnostic investigation, such as age, comorbidities, performance status, localisation of metastasis, or the patient’s decision. The second sensitivity analysis was performed after the first 2 years of follow-up had been excluded so as to check for potential reverse causality bias as a result of preclinical cancer at baseline. Reverse causality bias may occur if participants change their dietary behaviour as a result of symptoms of preclinical cancer, whereas we are interested to see if dietary behaviour reduces or increases CUP risk. In the third sensitivity analysis, the first 10 years of follow-up (< 1996) were compared to the last 10 years of follow-up (≥ 1996), as to see whether associations between meat consumption and CUP risk differed over time.

## Results

The statistical analyses of this study are based on 899 incident CUP cases and 4111 subcohort members with complete and consistent dietary data.

CUP cases appeared to consume slightly more red meat (overall), processed meat, and fish than subcohort members (90.8 g/day vs. 86.9 g/day and 15.0 g/day vs. 13.1 g/day and 14.1 g/day vs. 12.9 g/day, respectively) (Table 1). By contrast, subcohort members ate slightly more poultry than CUP cases (13.5 g/day vs. 12.9 g/day). The comparison between CUP cases and subcohort members appeared to be confounded by sex with respect to consuming red meat (overall) and processed meat. Male CUP cases consumed more red meat (overall) than male subcohort members (95.1 g/day vs. 93.4 g/day). Female CUP cases consumed more red meat (overall) than female subcohort members (83.5 g/day vs. 80.6 g/day). In addition, male CUP cases ate slightly more processed meats than male subcohort members (16.4 g/day vs. 15.8 g/day). Female CUP cases also ate more processed meats than female subcohort members (12.7 g/day vs. 10.4 g/day). Neither poultry consumption nor fish consumption appeared to be confounded by sex.

**Table 1:** Characteristics of cancer of unknown primary cases and subcohort members in The Netherlands cohort study

Characteristic	Subcohort members (*n* = 4111)		Cancer of Unknown Primary cases (*n* = 899)	
	*n*	(%)	*n*	(%)
Age at baseline (years)				
55–59	1605	39.0	276	30.7
60–64	1402	34.1	349	38.8
65–69	1104	26.9	274	30.5
Sex				
Men	2022	49.2	568	63.2
Women	2089	50.8	331	36.8
Red meat consumption (g/day)	86.9 (40.5)		90.8 (40.3)	
Men	93.4 (41.6)		95.1 (41.6)	
Women	80.6 (38.3)		83.5 (37.0)	
Processed meat consumption (g/day)	13.1 (14.6)		15.0 (15.5)	
Men	15.8 (16.7)		16.4 (15.6)	
Women	10.4 (11.6)		12.7 (15.0)	
Poultry consumption (g/day)	13.5 (15.1)		12.9 (14.4)	
Men	13.6 (14.7)		13.3 (15.3)	
Women	13.4 (15.4)		12.2 (12.8)	
Fish consumption (g/day)	12.9 (15.3)		14.1 (17.9)	
Men	14.1 (16.6)		15.5 (20.2)	
Women	11.7 (13.9)		11.7 (12.8)	
Ethanol intake (g/day)^a^				
Abstainers	975	23.7	170	18.9
< 5	1179	28.7	233	25.9
5– < 15	938	22.8	204	22.7
15– < 30	651	15.8	143	15.9
≥ 30	368	9.0	149	16.6
Cigarette smoking status				
Never smokers	1517	36.9	249	27.7
Ex-smokers	1479	36.0	317	35.3
Current smokers	1115	27.1	333	37.0
Frequency of cigarette smoking (*N*/day), mean (SD)^b^	15.7 (10.1)		17.9 (10.4)	
Duration of cigarette smoking (years), mean (SD)^b^	31.8 (12.1)		35.4 (11.7)	
Body mass index at baseline (kg/m^2^), mean (SD)	25.0 (3.1)		24.9 (3.0)	
Non-occupational physical activity (min/day)				
≤ 30	844	20.8	182	20.5
> 30–60	1269	31.2	274	30.9
> 60–90	857	21.1	160	18.0
> 90	1093	26.9	271	30.6
Level of education (years of education)				
Primary	1154	28.2	237	26.6
Lower vocational	899	22.0	192	21.6
Secondary and medium vocational	1457	35.6	329	36.9
University and higher vocational	582	14.2	133	14.9
Diabetes				
Yes	140	3.4	36	4.0
First grade family history of cancer				
Yes	1870	45.5	436	48.5

^a^In consumers only

^b^In users only

Findings of the age and sex adjusted analyses were comparable to those of the multivariable adjusted analyses, which were additionally adjusted for alcohol consumption, cigarette smoking variables (status, frequency, duration), and total energy intake. Hence, only the results of the multivariable analyses are described below. In general, we observed no association between red meat (overall) consumption and CUP risk (HR for Q4 vs. Q1 1.04, 95% CI 0.83–1.30, *P*_trend_ = 0.31) (Table 2). We observed an increased risk between beef consumption and CUP, for which a statistically significant trend was found (HR for Q4 vs. Q1 1.22, 95% CI 0.99–1.52, *P*_trend_ = 0.02). A statistically significant association was also observed between processed meat consumption and CUP risk (HR for Q4 vs. Q1 1.40, 95% CI 1.12–1.75, *P*_trend_ = 0.006). No association was found between poultry consumption and CUP risk (HR for C4 vs. C1 0.97, 95% CI 0.79–1.21, *P*_trend_ = 0.28). For fish consumption, we observed an increased CUP risk, but it was not statistically significant (HR for Q4 vs. Q1 1.25, 95% CI 0.99–1.57, *P*_trend_ = 0.29).

**Table 2:** Hazard ratios and 95% confidence intervals for meat consumption and cancer of unknown primary risk in the Netherlands cohort study

			Subcohort members (*n* = 4111)	Cancer of Unknown Primary cases (*n* = 899)				
	Categorical median (grams per day)		Person time at risk (years)	Cases	Age and sex adjusted^a^		Multivariable adjusted^b^	
	Men	Women		*n*	HR	95% CI	HR	95% CI
Red meat (overall)								
Q1	50	41	17 433	205	1	Reference	1	Reference
Q2	79	68	17 338	224	1.14	(0.92–1.41)	1.11	(0.89–1.37)
Q3	100	88	17 184	248	1.26	(1.02–1.55)	1.21	(0.98–1.49)
Q4	139	125	17 548	222	1.13	(0.91–1.40)	1.04	(0.83–1.30)
* p* for trend^c^					0.08		0.31	
Continuous, 50 g per day increments			69 503	899	1.08	(0.99–1.18)	1.05	(0.96–1.15)
Beef								
Q1	4	3	17 293	199	1	Reference	1	Reference
Q2	16	14	17 065	208	1.05	(0.84–1.30)	1.03	(0.82–1.28)
Q3	30	26	17 986	231	1.10	(0.89–1.36)	1.08	(0.87–1.34)
Q4	53	47	17 160	261	1.25	(1.01–1.54)	1.22	(0.99–1.52)
* p* for trend^c^					0.01		0.02	
Continuous, 50 g per day increments			69 503	899	1.21	(1.04–1.41)	1.21	(1.03–1.42)
Pork								
Q1	9	5	17 461	214	1	Reference	1	Reference
Q2	28	23	17 308	250	1.21	(0.98–1.48)	1.14	(0.93–1.41)
Q3	44	40	17 288	216	1.07	(0.86–1.32)	1.01	(0.81–1.25)
Q4	74	65	17 445	219	1.09	(0.88–1.35)	0.99	(0.79–1.23)
* p* for trend^c^					0.65		0.78	
Continuous, 50 g per day increments			69 503	899	1.03	(0.91–1.16)	0.98	(0.86–1.12)
Minced meat								
Q1	3	0	16 932	237	1	Reference	1	Reference
Q2	12	10	17 106	239	1.01	(0.82–1.24)	1.01	(0.82–1.24)
Q3	21	18	17 801	207	0.87	(0.70–1.07)	0.86	(0.70–1.07)
Q4	38	33	17 664	216	0.90	(0.73–1.11)	0.91	(0.74–1.13)
* p* for trend^c^					0.23		0.22	
Continuous, 50 g per day increments			69 503	899	0.87	(0.69–1.09)	0.86	(0.68–1.09)
Liver								
C1	0	0	44 786	585	1	Reference	1	Reference
C2	4	3	24 716	314	0.99	(0.84–1.16)	0.96	(0.82–1.12)
* p* for trend^c^					0.92		0.87	
Continuous, 25 g per day increments			69 503	899	1.02	(0.65–1.63)	0.96	(0.59–1.56)
Poultry								
C1	0	0	16 123	203	1	Reference	1	Reference
C2	5	5	17 045	230	1.12	(0.90–1.38)	1.13	(0.91–1.41)
C3	13	13	16 570	227	1.12	(0.90–1.38)	1.10	(0.88–1.37)
C4	23	23	19 766	239	0.98	(0.79–1.21)	0.97	(0.79–1.21)
* p* for trend^c^					0.31		0.28	
Continuous, 50 g per day increments			69 503	899	0.88	(0.68–1.13)	0.86	(0.66–1.13)
Processed meat								
Q1	1	0	17 060	201	1	Reference	1	Reference
Q2	8	4	17 949	216	1.09	(0.88–1.35)	1.07	(0.86–1.33)
Q3	16	11	17 095	222	1.16	(0.93–1.44)	1.14	(0.91–1.42)
Q4	33	22	17 398	260	1.38	(1.12–1.70)	1.40	(1.12–1.75)
* p* for trend^c^					0.006		0.006	
Continuous, 25 g per day increments			69 503	899	1.16	(1.04–1.30)	1.19	(1.05–1.34)
Fish								
Q1	0	0	19 848	208	1	Reference	1	Reference
Q2	5	5	15 592	216	1.29	(1.04–1.59)	1.30	(1.04–1.61)
Q3	15	15	21 045	286	1.26	(1.03–1.54)	1.23	(1.00–1.51)
Q4	32	28	13 018	189	1.32	(1.06–1.65)	1.25	(0.99–1.57)
* p* for trend^c^					0.08		0.29	
Continuous, 25 g per day increments			69 503	899	1.11	(0.99–1.24)	1.07	(0.95–1.20)

^a^Analyses were adjusted for age at baseline (years) and sex

^b^Analyses were adjusted for age at baseline (years), sex, alcohol consumption, cigarette smoking status (never/ever), cigarette smoking frequency (continuous; centered), cigarette smoking duration (continuous; centered), and total energy intake (kcal/day)

^c^Tests for dose–response trends were assessed by fitting ordinal variables as continuous terms in the Cox proportional hazards model

As described above, meat consumption differed markedly between men and women concerning both red meat (overall) and processed meat. Therefore, we stratified the analyses based on sex (Table 3). For beef consumption and CUP risk in men alone, the association attenuated and the trend was no longer statistically significant (HR for Q4 vs. Q1 1.12, 95% CI 0.85–1.47, *P*_trend_ = 0.31). Conversely, for beef consumption and CUP risk in women alone, the association became stronger and was statistically significant (HR for Q4 vs. Q1 1.47, 95% CI 1.04–2.07, *P*_trend_ = 0.004). For processed meat consumption and CUP risk in men alone, the association slightly attenuated and was no longer statistically significant (HR for Q4 vs. Q1 1.33, 95% CI 0.99–1.79, *P*_trend_ = 0.15). Yet, the association appeared to be more pronounced in women and remained statistically significant (HR for Q4 vs. Q1 1.53, 95% CI 1.08–2.16, *P*_trend_ = 0.001).

**Table 3:** Hazard ratios and 95% confidence intervals for meat consumption and Cancer of Unknown Primary risk in the Netherlands Cohort Study, stratified for sex

	Men only					Women only				
	Categorical median (grams per day)	Subcohort members (*n* = 2022)	Cancer of Unknown Primary cases (*n* = 568)			Categorical median (grams per day)	Subcohort members (*n* = 2089)	Cancer of Unknown Primary cases (*n* = 331)		
		Person time at risk (years)	Cases	Multivariable adjusted^a^			Person time at risk (years)	Cases	Multivariable adjusted^a^	
			*n*	HR	95% CI			*n*	HR	95% CI
Red meat (overall)										
Q1	50	8 143	135	1	Reference	41	9 290	70	1	Reference
Q2	79	7 692	142	1.07	(0.81–1.42)	68	9 376	82	1.19	(0.85–1.68)
Q3	100	7 856	146	1.10	(0.83–1.44)	88	9 328	102	1.43	(1.03–2.00)
Q4	139	8 222	145	1.01	(0.76–1.35)	125	9326	77	1.09	(0.77–1.54)
* p* for trend^b^				0.70					0.20	
Continuous, 50 g per day increments		32 182	568	1.02	(0.91–1.16)		37 320	331	1.10	(0.95–1.27)
Beef										
Q1	4	7 988	133	1	Reference	3	9 305	66	1	Reference
Q2	16	8 003	134	0.97	(0.73–1.29)	14	9 062	74	1.15	(0.81–1.65)
Q3	30	8 116	138	0.97	(0.73–1.29)	26	9 870	93	1.34	(0.95–1.88)
Q4	53	8 076	163	1.12	(0.85–1.47)	47	9 083	98	1.47	(1.04–2.07)
* p* for trend^b^				0.31					0.004	
Continuous, 50 g per day increments		32 182	568	1.11	(0.91–1.37)		37 320	331	1.41	(1.11–1.78)
Pork										
Q1	9	8 157	128	1	Reference	5	9 305	86	1	Reference
Q2	28	8 037	151	1.13	(0.85–1.49)	23	9 271	99	1.16	(0.85–1.59)
Q3	44	7 787	137	1.07	(0.80–1.43)	40	9 502	79	0.90	(0.64–1.25)
Q4	74	8 202	152	1.15	(0.86–1.53)	65	9 244	67	0.75	(0.53–1.06)
* p* for trend^b^				0.49					0.10	
Continuous, 50 g per day increments		32 182	568	1.06	(0.90–1.25)		37 320	331	0.83	(0.66–1.04)
Minced meat										
Q1	3	7 829	162	1	Reference	0	9 103	75	1	Reference
Q2	12	8 049	151	0.92	(0.70–1.20)	10	9 056	88	1.20	(0.86–1.68)
Q3	21	8 138	127	0.76	(0.58–1.00)	18	9 663	80	1.07	(0.76–1.52)
Q4	38	8 166	128	0.78	(0.59–1.03)	33	9 498	88	1.19	(0.85–1.67)
* p* for trend^b^				0.07					0.53	
Continuous, 50 g per day increments		32 182	568	0.76	(0.56–1.03)		37 320	331	1.13	(0.77–1.64)
Liver										
C1	0	19 695	362	1	Reference	0	25 092	223	1	Reference
C2	4	12 488	206	0.92	(0.75–1.12)	3	12 229	108	1.03	(0.80–1.33)
* p* for trend^b^				0.88					0.98	
Continuous, 25 g per day increments		32 182	568	0.95	(0.52–1.75)		37 320	331	0.99	(0.45–2.19)
Poultry										
C1	0	7 542	121	1	Reference	0	8 580	82	1	Reference
C2	5	7 248	151	1.33	(1.00–1.76)	5	9 797	79	0.87	(0.62–1.21)
C3	13	8 044	139	1.08	(0.81–1.44)	13	8 526	88	1.14	(0.82–1.59)
C4	23	9 348	157	1.07	(0.80–1.41)	23	10 417	82	0.84	(0.60–1.17)
* p* for trend^b^				0.87					0.11	
Continuous, 50 g per day increments		32 182	568	0.97	(0.67–1.40)		37 320	331	0.73	(0.50–1.07)
Processed meat										
Q1	1	8 075	135	1	Reference	0	8 986	66	1	Reference
Q2	8	7 974	126	0.98	(0.74–1.30)	4	9 975	90	1.25	(0.88–1.76)
Q3	16	8 097	149	1.15	(0.87–1.53)	11	8 998	73	1.12	(0.78–1.61)
Q4	33	8 036	158	1.33	(0.99–1.79)	22	9 362	102	1.53	(1.08–2.16)
* p* for trend^2^				0.15					0.001	
Continuous, 25 g per day increments		32 182	568	1.11	(0.96–1.28)		37 320	331	1.50	(1.17–1.93)
Fish										
Q1	0	8 231	119	1	Reference	0	11 617	89	1	Reference
Q2	5	7 256	144	1.36	(1.02–1.81)	5	8 336	72	1.18	(0.84–1.66)
Q3	15	10 054	169	1.12	(0.85–1.48)	15	10 991	117	1.43	(1.05–1.95)
Q4	32	6 642	136	1.31	(0.98–1.76)	28	6 377	53	1.10	(0.76–1.61)
* p* for trend^2^				0.22					0.99	
Continuous, 25 g per day increments		32 182	568	1.10	(0.95–1.27)		37 320	331	1.00	(0.82–1.21)

^a^Analyses were adjusted for age at baseline (years), alcohol consumption, cigarette smoking status (never/ever), cigarette smo frequency (continuous, centered), cigarette smoking duration (continuous, centered), and total energy intake (kcal/day)

^b^Tests for dose–response trends were assessed by fitting ordinal variables as continuous terms in the Cox proportional hazards model

Furthermore, we checked whether there was a potential for residual confounding by cigarette smoking status and the association between meat consumption and CUP risk. We observed that the associations between beef and processed meat consumption and CUP risk increased when comparing current smokers to never smokers in women (data not shown). It should, however, be acknowledged that there were fewer cases available in the categories due to the stratification for both sex and cigarette smoking status. Our observations suggest that residual confounding by cigarette smoking status is unlikely in women.

We observed no multiplicative interactions between sex and the consumption of red meat (overall), beef, pork, minced meat, liver, processed meat, poultry, or fish in relation to CUP risk (*P*_interaction_ = 0.64, 0.55, 0.22, 0.19, 0.41, 0.52, 0.11, and 0.22, respectively). In addition, no multiplicative interactions were observed between cigarette smoking status and the consumption of red meat (overall), beef, pork, minced meat, liver, processed meat, poultry, or fish in relation to CUP risk (*P*_interaction_ = 0.27, 0.88, 0.22, 0.56, 0.14, 0.24, 0.88, and 0.80, respectively).

Results from the first sensitivity analysis with restriction to histologically verified CUP cases alone, for whom extended diagnostic methods are more expected (compared to cytologically verified CUP cases), indicate that the findings are similar to those of the overall multivariable analyses except for beef consumption and CUP risk (HR for Q4 vs. Q1 1.16, 95% CI 0.91–1.49, *P*_trend_ = 0.21), possibly due to fewer cases. We presume that the results of the overall multivariable analyses represent CUP cases with or without an extensive diagnostic work-up. Our secondary sensitivity analysis, in which the first 2 years of follow-up were excluded so as to check for potential reverse causality bias, also demonstrate similar findings to those observed in the complete analysis (data not shown). In our third sensitivity analysis, after splitting the follow-up time to compare the first 10 years of follow-up to the last 10 years of follow-up, we observed that the association between beef consumption and CUP risk was the highest in the first 10 years of follow-up, whereas it attenuated in the last 10 years of follow-up. On the other hand, for processed meat consumption and CUP risk, no association was found in the first 10 years of follow-up, while there was a positive statistically significant association in the last 10 years of follow-up.

## Discussion

In this detailed investigation of meat consumption and CUP risk, we found that beef and processed meat consumption were positively associated with the development of CUP in women. We found a non-significant positive association between processed meat consumption and CUP risk in men. In contrast, no associations were observed between red meat (overall), poultry, or fish consumption and CUP risk. We observed no multiplicative interactions between sex or cigarette smoking status and meat consumption and CUP risk.

To the best of our knowledge, only one study has previously investigated the relationship between red meat and processed meat and CUP risk. The abovementioned Australian cohort study compared 327 incident CUP cases to two sets of controls (3:1) that were randomly selected using incidence density sampling with replacement. Their study found no relation between red meat consumption and CUP risk, it used the usual number of servings as > 3 red meats/week compared to < 3 red meats/week for dichotomous comparisons [11]. For processed meat consumption and CUP, its authors observed an increased risk when comparing the usual number of servings as > 3 processed meats/week compared to < 3 processed meats/week, although the association was not statistically significant [11]. In the NLCS, by contrast, we have investigated the association between meat consumption and CUP risk in greater detail by assessing combined groups of meats, such as red meat, processed meat, poultry, and fish, as well as individual meat items. We have found that beef and processed meat consumption are significantly associated with an increased CUP risk, but that red meat (overall), poultry, and fish consumption do not appear to be associated with CUP risk. Consequently, while our study confirms the findings of the Australian cohort study in indicating no association between red meat (overall) consumption and CUP risk, we do observe an association between beef and processed meat consumption and CUP risk [11]. The consumption of red and processed meat has been linked to colorectal cancer in previous epidemiological studies (probable increasing risk and convincing increasing risk, respectively) [26]. It also known that colorectal cancer predominantly metastasises to the liver via the portal circulation [27]; therefore, we have conducted an additional analysis to study whether the association between meat consumption is stronger in CUP patients with metastases located in the liver. We found the association between processed meat consumption and CUP risk in patients with a liver metastasis to be increased (per 25 g per day increment HR 1.34, 95% CI 1.14–1.58, *P*_trend_ = 0.001) compared to the result of the overall analysis (per 25 g per day increment HR 1.19, 95% CI 1.05–1.34, *P*_trend_ = 0.006). In addition, based on data obtained from the NCR, 36.1% of the primary tumours that metastasised to the liver, originated in the colorectum (ICD-O-3 C18-C20) between 1986 and 2006 in the Dutch population. In line with the results of our analysis, it is thus plausible that in a considerable number of CUP patients with a liver metastasis, the primary tumour origin is the colorectum. Furthermore, we have checked the potential of residual confounding by cigarette smoking status. Despite studying fewer cases in the categories of interest due to stratification based on sex and cigarette smoking status, the association between beef and processed meat consumption did not differ greatly between the strata (never, ex, current smokers) in women, thereby hinting that the potential of residual confounding is unlikely. We have also checked whether splitting the follow-up time had an influence on the association between meat consumption and CUP risk. We observed that the association between beef consumption and CUP risk was highest in the first 10 years of follow-up, whereas it attenuated in the last 10 years of follow-up. For processed meat consumption and CUP risk, no association was found in the first 10 years of follow-up, while there was a positive statistically significant association in the last 10 years of follow-up. An indication for these findings might be that there is a shorter latency period for beef consumption and a relatively longer latency period for processed meat consumption, or that it concerns a chance finding as there were fewer cases available due to splitting the follow-up time. Therefore, more studies would be needed to investigate such conclusions.

As briefly presented in the introduction, scientific evidence has already revealed associations between red meat intake and processed meat intake and the development of specific cancers, though the associations are less consistent concerning poultry and fish consumption and carcinogenesis [13, 17]. As we have demonstrated here, however, there does appear to be a discernible connection between the consumption of beef and processed meats and the development of CUP (Table 4).

**Table 4:** Hazard ratios and 95% confidence intervals for meat consumption and cancer of unknown primary risk in the Netherlands cohort study, stratified for cigarette smoking status

	Smoking status = never						Smoking status = ex							Smoking status = current						
	Subcohort members (*n*=1517)	Cancer of Unknown Primary cases (*n*=249)					Subcohort members (*n*=1479)		Cancer of Unknown Primary cases (*n*=317)					Subcohort members (*n*=1115)	Cancer of Unknown Primary cases (*n*=333)					
	Person time at risk (years)	Cases	Age and sex adjusted^a^		Multivariable adjusted^b^		Person time at risk (years)		Cases	Age and sex adjusted^a^		Multivariable adjusted^b^		Person time at risk (years)	Cases	Age- and sex-adjusted^a^		Multivariable adjusted^b^		
		*n*	HR	95% CI	HR	95% CI			*n*	HR	95% CI	HR	95% CI		*n*	HR	95% CI	HR	95% CI	p for interaction^c^
Red meat (overall)																				0.273
Q1	7 381	59	1	Reference	1	Reference	6 067		73	1	Reference	1	Reference	3 895	73	1	Reference	1	Reference	
Q2	6 603	71	1.39	(0.95–2.02)	1.39	(0.95–2.04)	6 476		84	1.15	(0.81–1.63)	1.15	(0.81–1.65)	4 259	69	0.88	(0.60–1.29)	0.84	(0.57–1.24)	
Q3	6 891	74	1.40	(0.96–2.04)	1.43	(0.98–2.08)	6 071		77	1.08	(0.76–1.55)	1.00	(0.70-1.45)	4 221	97	1.27	(0.88–1.82)	1.22	(0.84–1.75)	
Q4	6 403	45	0.93	(0.61–1.42)	0.96	(0.62–1.48)	6 134		83	1.18	(0.83–1.69)	1.01	(0.69–1.47)	5 011	94	1.09	(0.76-1.57)	1.05	(0.72–1.53)	
* p* for trend^d^			0.74		0.59					0.67		0.56				0.11		0.12		
Continuous, 50 g per day increments	27 278	249	1.03	(0.88–1.20)	1.05	(0.89–1.23)	24 749		317	1.03	(0.89–1.19)	0.95	(0.81–1.12)	17 477	333	1.14	(0.97–1.34)	1.15	(0.97–1.36)	0.329
Beef																				0.880
Q1	6 475	48	1	Reference	1	Reference	6 205		70	1	Reference	1	Reference	4 612	81	1	Reference	1	Reference	
Q2	6 859	64	1.23	(0.82–1.85)	1.24	(0.83–1.85)	5 929		67	1.03	(0.71–1.49)	1.01	(0.69–1.48)	4 277	77	0.94	(0.65–1.36)	0.90	(0.62–1.31)	
Q3	7 221	70	1.30	(0.87–1.94)	1.31	(0.88–1.95)	6 227		79	1.09	(0.76–1.56)	1.08	(0.75–1.56)	4 487	82	0.98	(0.68–1.40)	0.94	(0.65–1.36)	
Q4	6 722	67	1.23	(0.81–1.84)	1.25	(0.83–1.88)	6 337		101	1.36	(0.97–1.93)	1.29	(0.90–1.83)	4 100	93	1.18	(0.83–1.69)	1.15	(0.80–1.67)	
* p* for trend^d^			0.25		0.21					0.05		0.12				0.18		0.18		
Continuous, 50 g per day increments	27 278	249	1.19	(0.89–1.59)	1.21	(0.90–1.63)	24 749		317	1.27	(1.00–1.60)	1.21	(0.95–1.54)	17 477	333	1.21	(0.91–1.61)	1.22	(0.91–1.63)	0.998
Pork																				0.220
Q1	7 206	67	1	Reference	1	Reference	6 488		79	1	Reference	1	Reference	3 768	68	1	Reference	1	Reference	
Q2	6 850	71	1.14	(0.79–1.64)	1.14	(0.79–1.65)	6 253		78	1.06	(0.74–1.50)	0.98	(0.69–1.40)	4 205	101	1.35	(0.93–1.94)	1.27	(0.88–1.84)	
Q3	6 731	67	1.13	(0.78–1.64)	1.14	(0.78–1.65)	5 944		78	1.16	(0.81–1.66)	1.11	(0.77–1.59)	4 614	71	0.85	(0.57–1.24)	0.82	(0.56–1.22)	
Q4	6 491	44	0.77	(0.51–1.17)	0.78	(0.52–1.19)	6 064		82	1.15	(0.81–1.64)	0.97	(0.67–1.41)	4 890	93	1.17	(0.81–1.70)	1.10	(0.75–1.62)	
* p* for trend^d^			0.03		0.04					0.73		0.63				0.19		0.24		
Continuous, 50 grams per day increments	27 278	249	0.77	(0.61– 0.97)	0.78	(0.61– 0.99)	24 749		317	1.04	(0.85– 1.27)	0.95	(0.76– 1.18)	17 477	333	1.15	0.93– 1.43)	1.15	(0.91– 1.44)	0.056
Minced meat																				0.560
Q1	6 896	60	1	Reference	1	Reference	5 702		86	1	Reference	1	Reference	4 334	91	1	Reference	1	Reference	
Q2	6 329	60	1.08	(0.73–1.60)	1.08	(0.74–1.60)	6 440		96	1.06	(0.76–1.47)	1.01	(0.72–1.42)	4 336	83	0.92	(0.65–1.31)	0.90	(0.63–1.29)	
Q3	7 369	65	1.09	(0.74–1.59)	1.09	(0.74–1.60)	6 183		63	0.72	(0.50–1.05)	0.70	(0.48–1.02)	4 248	79	0.87	(0.60–1.24)	0.86	(0.60–1.25)	
Q4	6 683	64	1.17	(0.79–1.71)	1.19	(0.80–1.75)	6 423		72	0.79	(0.55–1.12)	0.77	(0.54–1.10)	4 559	80	0.82	(0.58–1.17)	0.86	(0.60–1.24)	
* p* for trend^d^			0.32		0.29					0.03		0.02				0.46		0.61		
Continuous, 50 g per day increments	27 278	249	1.25	(0.80–1.95)	1.28	(0.82–2.01)	24 749		317	0.64	(0.43–0.95)	0.62	(0.41–0.93)	17 477	333	0.87	(0.59–1.27)	0.90	(0.62–1.33)	0.057
Liver																				0.140
C1	18 432	167	1	Reference	1	Reference	15 882		224	1	Reference	1	Reference	10 472	194	1	Reference	1	Reference	
C2	8 845	82	1.05	(0.78–1.40)	1.06	(0.79–1.43)	8 866		93	0.79	(0.60–1.04)	0.76	(0.58–1.01)	7 005	139	1.11	(0.85–1.44)	1.09	(0.83–1.42)	
* p* for trend^d^			0.34		0.29					0.06		0.06				0.81		0.81		
Continuous, 25 g per day increments	27 278	249	1.51	(0.65–3.52)	1.60	(0.68–3.77)	24 749		317	0.45	(0.19–1.03)	0.42	(0.17–1.02)	17 477	333	1.09	(0.56–2.10)	1.09	(0.55–2.15)	0.107
Poultry																				0.880
C1	6 611	58	1	Reference	1	Reference	5 505		70	1	Reference	1	Reference	4 006	75	1	Reference	1	Reference	
C2	6 856	65	1.12	(0.76–1.65)	1.13	(0.76–1.67)	5 754		72	1.06	(0.73–1.53)	1.07	(0.74–1.56)	4 434	93	1.13	(0.79–1.61)	1.21	(0.84–1.75)	
C3	6 156	62	1.19	(0.81–1.77)	1.21	(0.82–1.80)	6 113		80	1.13	(0.78–1.62)	1.13	(0.78–1.63)	4 301	85	1.04	(0.72–1.50)	1.02	(0.69–1.49)	
C4	7 654	64	0.99	(0.67–1.45)	1.00	(0.68–1.48)		7 376	95	1.11	(0.78–1.57)	1.09	(0.76–1.56)	4 735	80	0.86	(0.60–1.24)	0.88	(0.60–1.29)	
* p* for trend^d^			0.81		0.85					0.90		0.85				0.10		0.10		0.323
Continuous, 50 g per day increments	27 278	249	0.94	(0.58–1.52)	0.96	(0.59–1.54)		24 749	317	1.03	(0.68–1.54)	1.04	(0.67–1.61)	17 477	333	0.69	(0.44–1.07)	0.68	(0.43–1.07)	
Processed meat																				0.239
Q1	7 109	53	1	Reference	1	Reference		6 332	89	1	Reference	1	Reference	3 620	59	1	Reference	1	Reference	
Q2	6 710	70	1.49	(1.01–2.21)	1.52	(1.02–2.26)		6 661	66	0.76	(0.54–1.09)	0.77	(0.54–1.10)	4 578	80	1.15	(0.78-1.70)	1.12	(0.76–1.67)	
Q3	6 620	59	1.29	(0.86–1.94)	1.36	(0.89–2.07)		5 965	83	1.04	(0.74–1.47)	1.02	(0.72–1.44)	4 510	80	1.13	(0.77–1.67)	1.15	(0.77–1.71)	
Q4	6 838	67	1.42	(0.95–2.11)	1.55	(1.00–2.39)		5 791	79	1.09	(0.77–1.54)	1.06	(0.73–1.54)	4 769	114	1.53	(1.06–2.22)	1.66	(1.12–2.46)	
* p* for trend^d^			0.10		0.05					0.19		0.29				0.35		0.09		0.722
Continuous, 25 g per day increments	27 278	249	1.22	(0.96–1.56)	1.31	(1.00–1.71)		24 749	317	1.16	(0.93–1.44)	1.15	(0.89–1.48)	17 477	333	1.07	(0.92–1.25)	1.15	(0.98–1.36)	
Fish																				0.804
Q1	9 221	63	1	Reference	1	Reference		6 087	67	1	Reference	1	Reference	4 540	78	1	Reference	1	Reference	
Q2	5 865	59	1.56	(1.06–2.30)	1.58	(1.07–2.32)		5 983	79	1.13	(0.79–1.63)	1.12	(0.77–1.62)	3 744	78	1.22	(0.84–1.76)	1.26	(0.86–1.83)	
Q3	7 860	80	1.52	(1.06–2.18)	1.54	(1.07–2.22)		8 109	109	1.22	(0.87–1.71)	1.14	(0.80–1.62)	5 076	97	1.10	(0.78–1.56)	1.06	(0.74–1.53)	
Q4	4 331	47	1.57	(1.04–2.38)	1.63	(1.07–2.49)		4 570	62	1.20	(0.82–1.77)	1.16	(0.78–1.72)	4 117	80	1.13	(0.79–1.64)	1.06	(0.73–1.55)	
* p* for trend^d^			0.27		0.22					0.20		0.26				0.75		0.96		
Continuous, 25 g per day increments	27 278	249	1.12	(0.92–1.37)	1.14	(0.93–1.39)		24 749	317	1.14	(0.94–1.38)	1.12	(0.92–1.37)	17 477	333	1.03	(0.85–1.25)	0.99	(0.81–1.23)	0.659

^a^Analyses were adjusted for age at baseline (years) and sex

^b^Analyses were adjusted for age at baseline (years), sex, alcohol consumption, and total energy intake (kcal/day)

^c^Interactions were calculated with respect to smoking status in relation to the meat variable of interest and CUP risk

^d^Tests for dose–response trends were assessed by fitting ordinal variables as continuous terms in the Cox proportional hazards model

### Strengths and limitations

Important strengths of this study are its prospective cohort design, large sample size of 1,20,852 participants, large number of incident CUP cases, and the detailed availability of exposure and confounder data. Moreover, completeness of record linkage with the NCR and PALGA for cancer follow-up was at least 96%, which minimizes selection bias [28]. Vital status follow-up was complete for almost 100% after 20.3 years. Details on incident CUP cases were obtained from the NCR and included specific information from both pathology reports and clinical reports [29]. In addition, we could access the pathology excerpts and thus check whether the cytological and/or histological confirmed cases had been correctly categorised in the data provided by the NCR. The NCR registry clerks applied uniform coding rules when entering data based on medical files.

There are certain limitations that should be acknowledged. Exposure data on meat consumption were only measured at baseline in 1986, so participants may have changed their dietary habits after having completed the questionnaire, which could result in bias due to misclassification. The questionnaire was tested, however, both for validity and reproducibility purposes and appeared to be representative for dietary habits over a period of at least 5 years [22, 23]. In addition, this potential bias should be non-differential between CUP cases and subcohort members.

## Conclusions

Beef and processed meat consumption appear to be positively associated with CUP risk in women. Similarly, a positive association was found between processed meat consumption and CUP risk in men, although it was not statistically significant. We found no associations between red meat (overall), poultry, or fish consumption and CUP risk.

## Supplementary Information

Below is the link to the electronic supplementary material.

Supplementary file1 (XLSX 17 KB)

Supplementary file2 (XLSX 20 KB)

## Data Availability

The data sets generated and/or analysed during the current study are not publicly available, because the informed consent does not allow for that. However, anonymous data that are minimally required to replicate the outcomes of the study will be made available upon reasonable request and approval by the institutional review boards.

## Abbreviations

CI: Confidence interval
CUP: Cancer of unknown primary
HR: Hazard ratio
NCR: Netherlands cancer registry
NLCS: Netherlands cohort study on diet and cancer
PALGA: Dutch pathology registry
